# Coping with dementia caregiving: a mixed-methods study on feasibility and benefits of a psycho-educative group program

**DOI:** 10.1186/s12877-018-0896-y

**Published:** 2018-09-10

**Authors:** S. Pihet, S. Kipfer

**Affiliations:** 1 School of Health, University of Applied Sciences and Arts Western Switzerland, Fribourg, Switzerland; 2 Haute Ecole de Santé Fribourg, Route des Cliniques 15, 1700 Fribourg, Switzerland

**Keywords:** Dementia, Informal caregiver, Psycho-educative intervention, Stress, Coping strategies

## Abstract

**Background:**

Persons with dementia experience a progressive decline associated with an increasing dependency. Most of the support they require to stay at home comes from their informal caregivers (IC). Dementia informal caregiving imposes high costs on IC’s health and quality of life, related to long periods of chronic stress. Based on evidence that more adequate coping strategies can reduce chronic stress and its negative consequences, and that psycho-educative interventions have the broadest effects on IC quality of life, the program “Learning to feel better… and help better” was developed in French-speaking Canada. This group intervention focusing on coping with the daily stress of dementia caregiving showed efficacy in decreasing the behavior problems of the person with dementia and the associated stress reactions in their IC. **The objective**s of our study were to examine within a one group pre- and post-test design 1) the feasibility of implementing the program in two regions of French-speaking Switzerland, 2) the effects of the program, and 3) the participants’ use of the trained strategies in daily life.

**Method:**

A mixed-methods concurrent nested design was used to quantitatively evaluate the feasibility, the effects on five core outcomes, and strategy use in daily life. Additional qualitative data documented in depth the acceptability and impact of the intervention.

**Results:**

We analyzed 18 complete data sets. Regarding feasibility, qualitative and quantitative results converged towards a very good acceptance of the program and a strong implication of the participants. Regarding effects, the program resulted in substantial and significant improvements in burden (*d* = 0.41, *p* < .05), psychological distress (*d* = 0.54, *p* < .05) and self-efficacy (*d* = 0.43, *p* < .05). The qualitative results emphasized the benefits of a group format: Participants felt understood by peers, could build new social bonds and experienced reduced social isolation. Data regularly collected in daily life showed that participants were using more and more over time the strategies they learned (*β*_*01*_ = 0.55, *p* < .001), particularly reframing.

**Conclusion:**

This study expands on the original one conducted by the developers of the program in French-speaking Canada, by showing the feasibility and the very promising effects of this intervention in two regions of French-speaking Switzerland.

## Background

Dementia is one of the main cause for disability and dependency in older people, with 46.8 million people affected worldwide in 2015. Due to population ageing, in the absence of a cure, we expect 75 million of persons living with dementia in 2030, and 132 million in 2050 [1]. In Switzerland, 148,000 persons were living with dementia in 2016, and this number is expected to double in 2040, due to the Swiss population being among the oldest on the planet [2, 3].

Dementia is associated with a progressive decline in cognitive functions leading the affected person to depend more and more on the assistance, supervision and care of others. In Switzerland, more than half of the persons with dementia live at home (the other half in nursing homes), 43% requiring occasional support, 47% daily support, and 10% continuous support. Informal caregivers of the person with dementia (ICD), often their spouse or adult child, provide most of this support (e.g. [4, 5]). These figures are similar to those of other European countries (e.g. [4]).

The voluntary contribution of ICD is key to the sustainability of most health care systems (e.g. [6]). However taking an IC role is becoming more and more difficult as family size reduces, geographic distance between family members, employment rates of women, and professional pressures increase [7]. The sustainability of ICD contribution is a core public health issue. Supporting ICD is in the interest of the person receiving the care, the health care system and society in general. Describing the support options for IC in Switzerland is a challenge due to the fragmentation of this country composed of 26 states with substantial independence in terms of health politics; Each state further comprises several districts and a large number of municipalities, with some independence in terms of health care organization. Nevertheless, a nationwide evaluation of support options for ICD conducted in 2017 [8] revealed that two thirds of the states had homecare services with expertise in dementia in each of their districts (17, 65%). However only one-third had specialized daycare in each district (9, 35%) or a specialized coordination service (9, 35%), and only one-fourth had specialized night care services in each district (6, 23%). This overview focused primarily on publicly funded support options, while private associations (e.g. Alzheimer Association, Red Cross) also provide valuable offers such as support groups, adapted holidays or home respite, which again vary by state and district. In summary, key support options for IC of persons with dementia are available in most states of Switzerland. However there is a substantial heterogeneity across regions in their density, diversity, and level of coordination.

Despite the rewarding aspects of caregiving, such as maintaining continuity and closeness with the affected person (e.g. [9]), informal caregiving often imposes high demands and costs, particularly for those involved in dementia care. Many ICD experience long periods of chronic stress and heavy burden, reduced quality of life and social isolation, as well as more physical and mental health challenges, compared to their non-caregiving counterparts and to caregivers of persons without dementia (e.g. [5, 10]). ICD burden and health deterioration are core predictors of early institutionalisation [11] and mistreatment [12] of their care recipient.

### Learning to cope better in a psycho-educative group programs for ICD

How can we best prevent ICD exhaustion and protect their quality of life? Focusing on coping strategies with a psycho-educative intervention holds the most promise for countering their chronic stress. A recent meta-analysis of 56 multifactorial studies confirmed that coping strategies and self-efficacy were core and highly stable predictors of burden [13]. Two other meta-analyses showed that psycho-educative interventions have the broadest effects, compared to other forms of ICD support which mostly affect specific domains [14, 15]. Coping with the daily stress of dementia caregiving is the main focus of the psycho-educative program “Learning to feel better… and help better” developed in Quebec, Canada. This group intervention of 15 weekly sessions of 2 h each (for more detail see the methods section) is the sole intervention in French which has already been tested in a randomized controlled trial conducted across six centers. Comparing the 60 participants who received this intervention to 56 control ICD referred to support groups, the program was found more effective in decreasing the frequency of behavior problems of the person with dementia and the associated distress in ICD, with respective effect sizes of *d* = 0.09 and *d* = 0.38 [16]. To the best of our knowledge, such psycho-educative interventions are still seldom available in Switzerland [7]. Based on the above evidence providing support for the dissemination of this program for French-speaking ICD, we aimed to evaluate whether this program could be implemented in a different cultural context, namely French-speaking Switzerland, and to test if the developers’ results can be replicated in this new context. As little is yet known about the mechanisms of action of this program, documenting how participants use the strategies they learn in daily life can help gain some understanding of the change processes.

### Aims of the study

We aimed to examine within a one group pre- and post-test design 1) the feasibility of implementing the program in two regions of French-speaking Switzerland, 2) the effects of the program, and 3) the participants’ use of the trained strategies in daily life. We favored a mixed-methods approach with a concurrent nested design. We firstly aimed to collect quantitative evidence about feasibility (e.g. dropout rates, acceptability of different components of the program), effects on the five core outcomes considered in the developers’ study, and amount of strategy use in daily life. These five outcomes are the frequency of behavior problems of the person with dementia and the reactions of ICDs to these problems, as well as the ICDs’ subjective burden, self-efficacy, and psychological distress. In parallel, we aimed to obtain more in-depth qualitative information about the acceptability and impact of the intervention from the point of view of ICDs.

## Methods

In reporting on this quasi-experimental intervention study we follow the TIDieR guidelines [17]. The data presented here were collected from October 2014 to December 2015.

### Sample

We recruited a convenience sample of 26 ICD through service providers in the field of dementia (Alzheimer Association, home care nurses, memory clinics, daycare centers) operating in two regions of the French-speaking part of Switzerland. Participants volunteered for a free psycho-educative intervention focusing on stress management, along with pre- and post-intervention interviews and short reports on a tablet provided twice a week. Inclusion criteria were 1) being the primary caregiver of a person living with a diagnosis of dementia (as reported by the ICD based on a physician evaluation), 2) caring for this person since at least 6 months. Exclusion criteria were 1) insufficient French-language skills, 2) low caregiver burden (score below 10 on the Zarit Burden Interview), and 3) no patient memory and behavioral problems. Participating ICD were mostly women (73%, *N* = 19), spouses of the patient (69%, *N* = 18; others were children: 27%, *N* = 7; and siblings: 4%, *N* = 1), with a median age of 68 years (*Q1* = 60, *Q3* = 72, *range* 37–86). Patients were mostly men (58%, *N* = 15) with a median age of 77 years (*Q1* = 71, *Q3* = 82, *range* 56–94). All had a diagnosis of dementia including 50% (*N* = 13) Alzheimer, 39% (*N* = 10) unspecified or mixed, 4% (*N* = 1) vascular, 4% (*N* = 1) Lewy body, and 4% (*N* = 1) fronto-temporal. ICD predominantly lived in the same household as patients (81%, *N* = 21), had been providing care for a median duration of 3 years (*Q1* = 2, *Q3* = 5, *range* 0.5–10 years), and were currently in charge of the patient for a median of 6 days a week (*Q1* = 4, *Q3* = 7, *range* 1.5–7 days).

### Procedure

**Fig. 1:**
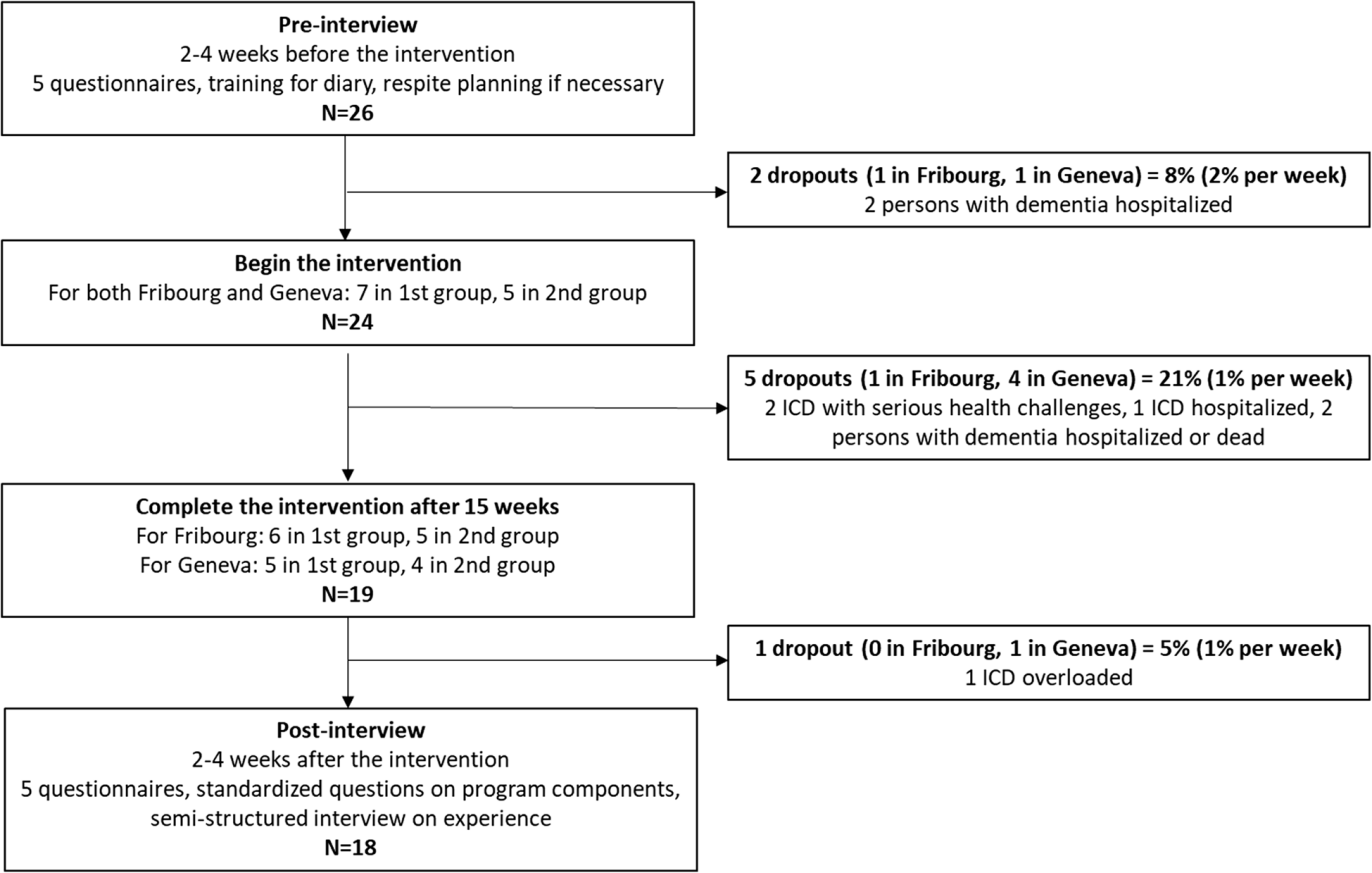
Flow chart

The study was performed in accordance with the declaration of Helsinki and approved by the local ethics review board (Commission cantonale (VD) d’éthique de la recherche sur l’être humain, protocol n°175/14). Written informed consent was obtained from each ICD after oral and written information provided by a member of the research team. Figure 1 offers an overview of the study procedure.

**Fig. 2:**
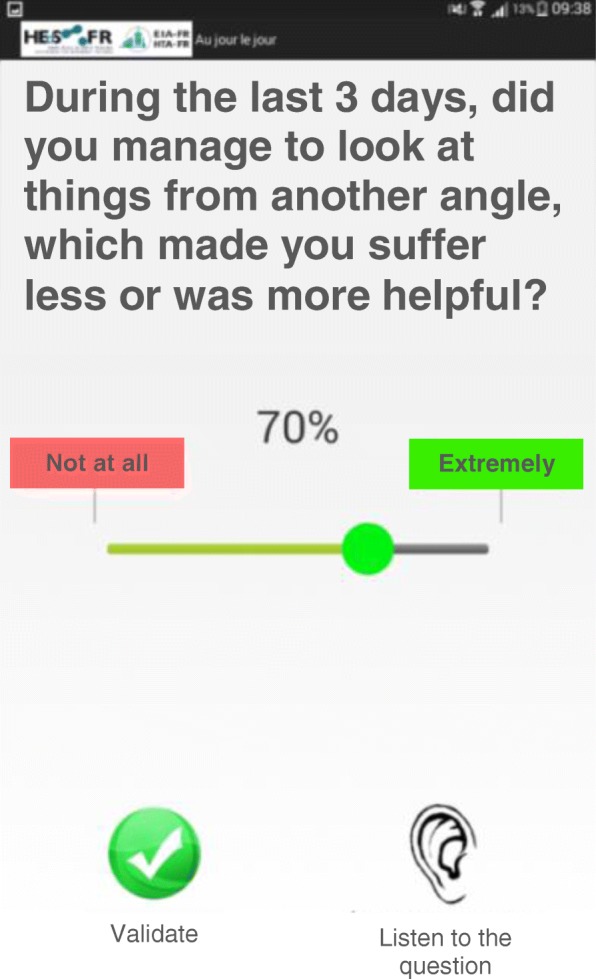
Example of a question (here for success in reframing) presented on the screen of the touchpad with the slider used to provide the answer

Before the intervention, during an individual interview with a trained researcher, participants completed five questionnaires and received training for the daily life reports on the electronic diary. The diary training involved an introduction to the handling of the touchpad (Samsung Galaxy Tab4) on which all daily life questions were answered, and the completion of the first data point with an explanation of each question. Each participant then received a touchpad to take home and was instructed to answer the daily life questions twice a week. By way of an application specifically developed for this study with a focus on usability for elderly, questions were presented one at a time on the screen of the touchpad, written in large characters, along with a slider to give the answer (see Fig. 2).

During the interview, we also discussed whether participants needed a volunteer to care for their loved one with dementia during the intervention, which we could organize with the local Alzheimer Association, our partner in the project. Participants then took part in the intervention described below. After the end of the intervention, participants took part in a second individual interview with the same researcher to complete the five questionnaires again, and report on their experience with the intervention on quantitative and qualitative questions.

### Intervention

The intervention was originally developed by Louise Levesque, Francine Ducharme and their team in the years 2000s based on Lazarus and Folkman transactional theory of stress and coping [18]. The program called “learning to feel better… and help better” aimed at improving the ability of ICD to cope with the stressful demands of caring for a person with dementia living at home [19]. The content of the program focuses on 1) the appraisal of stressful situations, and 2) the coping strategies. Regarding appraisal, participants learn to break down a global situation into specific ones, identify more precisely what is stressful, and distinguish between situations or aspects of it which can be modified and those which cannot. Regarding coping strategies, participants are trained to choose an appropriate strategy depending on whether the situation can be modified or not, use problem solving for modifiable situations (7-step procedure), use reframing for unmodifiable situations (look at things from another angle to reduce painful emotions), and seek for social support (identify precise support needs and best persons to address each of them). In addition, information is provided on how dementia may affect the communication and relational behavior of the affected person, and how ICD may improve their communication skills and prevent tensions. The program uses a combination of 1) information provision, 2) group discussions, 3) work on personal stressful situations, and 4) exercises at home. The content and methods used in program are described in details in Lévesque and co-authors [19]. Participants receive a booklet containing key information and exercise sheets, and the course leaders provide the intervention according to a detailed manual. The intervention consisted in 15 weekly group sessions of 2 h each, held in a quiet room at the School of Health in Fribourg or at a local daycare center in Geneva. We conducted two consecutive groups in Fribourg, and in Geneva as well. In both sites, 7 participants started the first program session and 5 started the second one. Two nurses with expertise in dementia and work with informal caregivers led the 15 sessions of each intervention group, with 5 different nurses involved across the 4 groups of participants. All nurses had completed a 4-day training to the intervention program and participated in 2 to 3 one-day supervision sessions organized throughout the intervention. The supervision was conducted by a trained psychologist and psychotherapist with extensive experience in psycho-educative group interventions. All sessions were audio-recorded and the adherence to the course manual was assessed as good for 4 randomly selected sessions for each site, by two independent coders who were trained to the program but were not involved in the study.

### Measures

We collected 1) *questionnaire measures* for five outcomes, 2) *daily life reports* for strategy use, and 3) *quantitative and qualitative post-intervention reports* on the experience in the program.

#### Questionnaire measures

##### Caregiver’s burden

We measured burden with the Zarit Burden Interview [20], a well-validated and widely used 22-items questionnaire. Responses are provided on a scale from 0 (*never*) to 4 (*very often*). Scores above 18 indicating an important burden and scores above 32 a severe one [21].

##### Memory and behavioral problems (MBP) and caregiver’s MBP-related distress

We measured these two outcomes with the Revised MBP Checklist [22], a questionnaire which measures the frequency of 24 MBP in the preceding week between 0 (*never*) and 4 (*daily*), and the extent to which this problem disturbed or upset the ICD between 0 (*not at all*) and 4 (*extremely*). This questionnaire is in French and has satisfactory psychometric properties: the factor structure is confirmed, the internal consistency is good and the convergent validity is well established [22].

##### Caregiver’s psychological distress

We used the short version of the Ilfeld Psychiatric Symptoms Index [23], a questionnaire asking participants to rate 14 symptoms related to depression, anxiety, anger and cognitive disturbance, on a 4-point scale from 1 (*never*) to 4 (*very often*). The psychometric properties are satisfactory for the English [23] and French [24] versions.

##### Caregiver’s self-efficacy

We measured self-efficacy as suggested by Bandura [25], with a visual analogue scale ranging from 0 (*no confidence at all in my ability to assume my caregiver role*) to 10 (*full confidence*).

#### Daily life measures

We collected daily life measures of participants’ use of the three strategies taught in the program throughout the 15-week intervention. As is typical in daily life studies, we used a limited number of questions to measure each construct, to prevent overloading the participants and to maintain compliance over time. The following questions were developed specifically for this study, focusing on the last 3 days, with answers provided on a visual analogue scale yielding a score between 0 (*not at all*) and 100 (*extremely*):

1. *Overall strategy use*. How much did you use the strategies you learned in the program?
2. *Problem solving.* Did you try to solve a problem for which you had no satisfactory solution? Did you manage to solve your problems satisfactorily?
3. *Reframing.* Did you notice some thoughts which made you suffer unnecessarily or exaggeratedly, or which did not help you? Did you try to question these thoughts? Did you manage to look at things from a different angle which made you suffer less or was more helpful?
4. *Support seeking.* Did you feel the need to receive support? Did you receive the support you needed? (An additional question was “Did you ask for the support you needed?”, but this piece of data was unusable due to a problem with the answer scale of the question).

#### Post-intervention reports on the experience in the program

##### Quantitative

We assessed the perceived usefulness of the 4 strategies taught in the program (communication, modifying unhelpful thoughts, problem solving and support seeking) and the 4 methods used (information provided by the course leaders, working on personal situations, group exchanges, and exercises at home) with 3 items for each: 1) I found it interesting, 2) I found it useful, and 3) It helped me in my daily life. The answers were given on a 5-point scale: 0 *Not at all or very little*, 1 *A little*, 2 *Moderately*, 3 *Very*, 4 *Extremely*. As they were highly correlated (in most cases Spearman’s ρ > .50) we averaged them. Regarding the 4 strategies, we also asked the two following questions: 1) I found it difficult to understand, 2) I found it difficult to apply, with answers given on the same 5-point scale.

##### Qualitative

Qualitative data was collected using semi-structured interviews. The interview guideline focused on the benefits of the program, negative aspects and the extent to which the program met the participants’ expectations. Participants were interviews in their homes 2 weeks after the end of the program. The interviews were audio recorded, transcribed word by word and anonymized.

### Data analysis

#### Quantitative

We used descriptive statistics to provide general information on the study outcomes (means and standard deviations, SD, to allow comparability with the original study, as well as median, Q1, Q3 as the variables showed some deviations from normality) and to analyze the quantitative post-intervention reports (median, Q1, Q3 and boxplots). The effects of the intervention on the five outcomes were tested with a repeated measures MANOVA, with time (pre versus post) and measure (the 5 questionnaires) as independent variables, and the score as the dependent variable. The effect of the intervention for each outcome was then tested with post hoc paired t-tests (one-tailed), and we computed effect sizes (Cohen’s d). Regarding strategy use in daily life, we assessed the mean level at the beginning of the intervention and the linear change over the course of the intervention with multilevel regression with full maximum likelihood estimation (using Hierarchical Linear Modeling, HLM 6; [26]), as the measurement occasions (within-person, Level 1, L1) were nested within the participants (Level 2, L2). Eq. 1 shows our model, where the use of each strategy is predicted by time in days (uncentered, i.e. coded 0 for the first day of the intervention) on L1. In this model *β*_*00*_ (i.e. the average intercept) indicates the average level of strategy use at the beginning of the intervention. *β*_*10*_ (i.e. the average slope) is used to test our hypotheses that strategy use increased over time, which is indicated by a positive slope (*p*s are thus one-tailed).

1 $$ {\displaystyle \begin{array}{l}\mathrm{Level}\ 1:\mathrm{Strategy}\ \mathrm{use}={\gamma}_0+{\gamma}_1\mathrm{Time}+\mathrm{E}\\ {}\mathrm{Level}\ 2:{\gamma}_0={\beta}_{00}+{R}_0\\ {}\kern6.5em {\gamma}_1={\beta}_{10}+{R}_1\end{array}} $$

#### Qualitative

The qualitative data was analysed using the approach from Mayring (2010) for summarizing content analyses with inductive category assignment. This approach is described as very fruitful to allow a true description and understanding of the original material “without bias owing to the preconceptions of the researcher” Mayring, 2014). After the definition of the material a first phase of paraphrasing and generalization of the data was performed. To develop a summarizing coding system, the material was further reduced and abstracted (first and second reduction according to Mayring, 2010). Finally, the summarizing coding system was re-checked with the original material to ensure that the category system matched it well. Findings and discrepancies in the interpretation of the data were regularly discussed in the research group. The analytical software Atlas.ti 7 assisted the analysis process.

## Results

### Feasibility

#### Recruitment process

This study was initiated in partnership with two core service providers in the field of dementia, the largest home care organization in the region of Geneva, and the local Alzheimer Association in the region of Fribourg. The Alzheimer Association also provided volunteers for the supervision of the person with dementia while the ICD attended the program in both regions. Initial efforts of the field partners to recruit through articles in the newsletter of the Alzheimer Association in Fribourg, and through the home care staff (using a flyer) in Geneva, brought only few participants. We then opted for a broader strategy by involving additional service providers (memory clinics, daycare centers, home care services in Fribourg as well) and organizing presentations of the program by the course leaders for each service provider. Regular articles were further published in the newsletters of the Alzheimer Association in Fribourg and the public hospital in Geneva. The main challenges in the recruitment process were: 1) Transporting the specificity of the program to the (often little trained) staff or volunteers involved in the recruitment, who repeatedly saw it as a mere support group; 2) Informing and screening the ICDs contacting us after reading an article or receiving a flyer, which often did not meet our rather stringent inclusion criteria or perceived the requirements of the program as too high in terms of duration, travel, or learning of skills; 3) Providing ICDs with information about the program at the right moment (when they were settled in their role but not yet exhausted), given by a trustworthy person or different independent sources (the memory clinic plus an article plus the home care nurse).

#### Dropout rates

Out of the 26 recruited participants, two dropped-out just before starting the program (both due to an unexpected physical illness of the person with dementia requiring hospitalization). Out of the 24 participants who started the program, 19 (79%) completed it. The five participants who dropped out did so within the first 6 sessions, due to hospitalization (after a serious fall, *N* = 1) or death (*N* = 1) of the person with dementia, or the hospitalization (after a serious fall, *N* = 1) or serious illness (*N* = 2) of the ICD. In addition, one participant was unable to complete the follow-up questionnaires due to overload at the time of data collection, so that 18 persons could be included in the final analyses.

#### Participation to the program sessions

The average participation rate in the program has been very high (92%). Nearly all participants (94%) took part in more than 12 sessions, to the exception of one ICD who missed 5 sessions due to professional obligations. The few other missed sessions were due to ICD sickness, the death of a relative, or an unavoidable appointment.

### Pre-test questionnaire results: Descriptives

The questionnaires filled in at pre-test (*N* = 26) showed that the ICD burden was overall severe (*M* = 39.56, *SD* = 14.21, *median* = 41.25, *Q1* = 31.00, *Q3* = 49.25), with a moderate frequency of patients’ MBP (*M* = 1.58, *SD* = 0.52, *median* = 1.62, *Q1* = 1.17, *Q3* = 1.88) and caregivers’ MBP-related distress (*M* = 1.86, *SD* = 0.66, *median* = 1.85, *Q1* = 1.35, *Q3* = 2.43). The ICD reported moderate psychological distress (*M* = 26.58, *SD* = 6.88, *median* = 26.50, *Q1* = 21.50, *Q3* = 29.00), and a rather high self-efficacy (*M* = 6.90, *SD* = 1.84, *median* = 7.50, *Q1* = 5.38, *Q3* = 8.50). As presented in Table 1, in comparison with the 8 persons who dropped out just before starting or during the program, the 18 completers had more pronounced difficulties on our five outcomes.

**Table 1:** Mean and (SD) for the study variables at pre-test and post-test

	Pre-test dropouts (*N* = 8)	Pre-test completers (*N* = 18)	Post-test (*N* = 18)	t-test pre-post (df = 17)	Effect size
Burden (0–88)	31.63 (17.6)	43.08 (11.3)	38.61 (10.7)	2.13*	0.41
MBP (0–4)	1.36 (0.6)	1.68 (0.5)	1.67 (0.6)	0.10	0.03
MBP-related distress (0–4)	1.58 (0.7)	1.98 (0.6)	1.98 (0.6)	−0.06	0.01
Psychological distress (14–56)	23.50 (8.3)	27.94 (5.9)	25.19 (4.4)	1.94*	0.54
Self-efficacy (0–10)	7.69 (1.6)	6.56 (1.9)	7.36 (1.9)	−2.33*	0.43

*Note.* For all the variables listed, higher scores indicate higher levels; *MBP* memory and behavioral problems; * *p* < .05, ** *p* < .01, *** *p* < .001 (one-tailed); for burden, scores above 18 indicate an important burden and scores above 32 a severe one

### Effects of the program: Changes in questionnaire scores

Comparing pre- and post-intervention scores (see Table 1) with a repeated measures MANOVA, we observed significant effects for time (*F*_(1,17)_ = 444.42, *p* < .001) and the time x measure interaction (*F*_(4,14)_ = 152.47, *p* < .001), indicating the intervention affected our outcomes though not all of them in the same way. Paired t-tests conducted post-hoc confirmed that burden and psychological distress decreased significantly, and self-efficacy increased significantly, with large effect sizes (see Table 1). There was no significant change in the frequency of patients’ MBP and caregivers’ MBP-related distress. We obtained highly similar results using non-parametric tests.

### Strategy use in daily life

At the beginning of the intervention, overall strategy use was moderate (*β*_*00*_ = 41.78 on a 0 to 100 scale). As hypothesized, overall strategy use increased substantially over time (*β*_*01*_ = 0.55, *p* < .001). Regarding problem solving, at program start, efforts to solve new problems were rather low (*β*_*00*_ = 31.35) however success in solving problems was rather high (*β*_*00*_ = 59.48). We found no significant change over time in efforts (*β*_*01*_ = − 0.01, *p* = .404) and a marginal increase in success (*β*_*01*_ = 0.10, *p* = .097). Regarding reframing, at the beginning of the intervention, the identification of unhelpful thoughts was rather low (*β*_*00*_ = 34.17), reframing efforts were moderate (*β*_*00*_ = 49.74), and success in reframing was rather high (*β*_*00*_ = 57.26). In line with expectations, we observed significant increases over time for the identification of unhelpful thoughts (*β*_*01*_ = 0.08, *p* = .035), efforts to reframe (*β*_*01*_ = 0.20, *p* = .006), and success at reframing (*β*_*01*_ = 0.25, *p* = .005). Regarding support seeking, at the beginning of the intervention, the need for support (*β*_*00*_ = 44.62), and success in obtaining it (*β*_*00*_ = 43.25) were moderate. Need for support increased marginally (*β*_*01*_ = 0.13, *p* = .062) while we found no significant change in obtaining support (*β*_*01*_ = − 0.02, *p* = .394). In summary, we observed most progresses in the use of reframing, along with a modest improvement in problem solving and a slightly increasing need for social support, but no systematic change in the support received.

### Satisfaction with the diverse methods and contents of the program

**Fig. 3:**
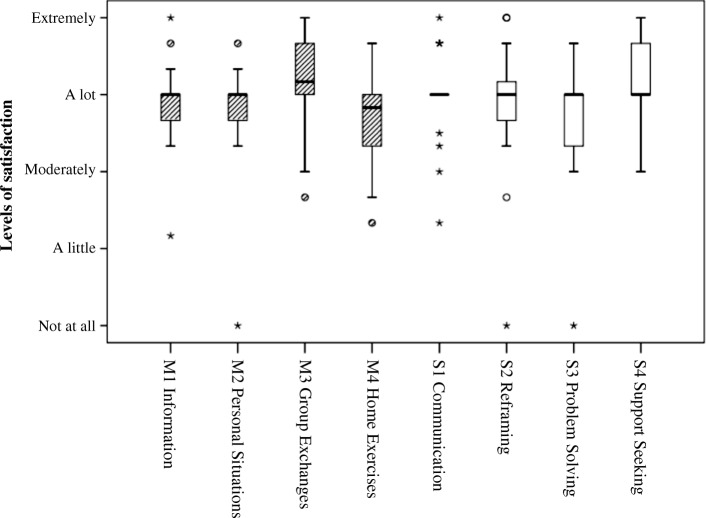
Satisfaction with methods (M) and strategies (S)

As presented in Fig. 3, the median value for the four methods used (information provided by the course leaders, working on personal situations, group exchanges, and exercises at home) was close to 3, corresponding to the answer “very relevant / useful / helpful in daily life”. The same was observed for the four types of strategies (communication, modifying unhelpful thoughts, problem solving and support seeking).

**Fig. 4:**
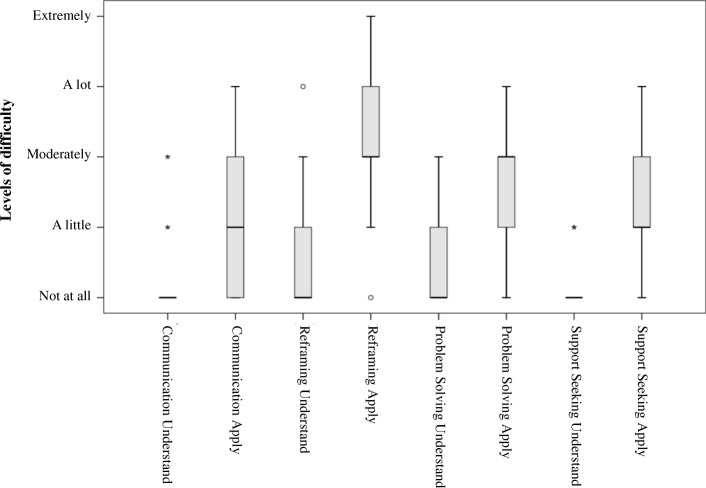
Perceived difficulty in understanding or applying the strategies

Figure 4 presents the results for the two questions exploring whether the participants perceived the four contents as difficult to understand or to apply. For the understanding all medians were at 0 (“Not at all”), and for the application all were between 1 (“A little”) and 2 (“Moderately”), the most challenging strategy being the modification of unhelpful thoughts.

### Benefits from and challenges in the program: Qualitative results

The content analysis revealed three main categories: 1) S*haring experiences and strategies*, which is about participants learning from each other, comparing their situations with others and getting strengthened in their way of handling the situation; 2) *Being in the same boat*, namely feeling less alone and feeling understood and connected to the other ICD as they are in similar situations; 3) *Being able to cope*, as the program empowered the participants to develop strategies which helped them managing their challenging situations.

#### Sharing experiences and strategies

The participants reported that they learned to talk about their situations and their experiences. For some it was the first time they had the confidence to talk about their difficult situation at home.

*«Being able to share and truly being listened to. For me the positive point was finding the confidence to express myself, to say what is going on with my husband. I now speak more often about it, yes, I can express myself more openly.»*

Sharing their experiences allowed them to reflect on their situation, compare it to the situations of others, and learn from each other, especially about helpful strategies.

*«It was a place, where I had room and time to reflect on everyday problems, to which I had no solution. It gave me the opportunity to learn strategies to solve these problems.»*

By comparing their situation with the experiences of other participants they also got encouraged in the way they handle some situations, they learned to appreciate their situation, for example when it was less challenging than those of others, and they got inspired by participants with particular helpful strategies.

*«So it made me finally take account of my situation, yes, it is not easy, there is no-one who wants this life, but I say to myself there are others who really are in much more complicated situations.»*

A participant, who was new to the caregiving role, felt enabled to anticipate and to set limits after listening to the experiences of exhaustion and heavy burden of other participants.

*«All the people who were there, some [people] had more painful and hard circumstances, so I said to myself, this is it, what I can expect for myself […]. It was very real. A bit too concrete, but this also allowed me to see the limits. How far I can go.»*

Listening to the experiences of others was not always described as an opportunity to learn. Some participants reported getting upset while listening to the burdening stories of others, or finding it painful to imagine they could be facing similar situations in the future.

*«Yes, I had a moment, two or three moments, where it was very hard for me during this course. It was perhaps when there were people who shared too much violent detail, which I wasn’t prepared for. That distressed me a bit. And then reality hits you.»*

#### Being in the same boat

Being in a group with other ICDs made the participants feel less alone. They realized that others shared the same problems or had similar difficult situations.

*«It brought me a fantastic well-being. Because you feel a bit lonely in the world, when you are like this, but then I saw, there are lots of people with the same problems as me.»*

The participants enjoyed feeling deeply understood by other caregivers with similar experiences or by group leaders with extensive knowledge in this field.

*«We need to talk about our experiences, and to talk to others who know and others who also share the same [experiences], even though they have no solutions, they share. Emotionally they can understand the burden of it, they can understand the degree of isolation that we can feel in this [situation].»*

Participants valued being in a small group, allowing a focus on the specific needs of each participant and discussions on practical issues related their specific situations.

*«It is clear, we were a small group, this also allowed us to develop the things we needed in parallel to the program. Suddenly we were a bit more than a program. It made the work valuable. We could really talk about practical cases […]. »*

The participants reported that they felt connected to the other participants and described feelings of friendship, empathy and solidarity. They appreciated and underlined the importance of their experience and that of others never being judged. Such a benevolent environment enabled the participants to build supportive relationships, which they expect to persist after the end of the program.

*«Me, I made friendships in the group. […] It is true, we will stay in contact. So we were not going to go our separate ways, on the contrary we exchanged our telephone numbers and all that and said we are going to meet regularly.»*

#### Being able to cope

This category shows the strongest connections to the content of the program and to the aim of empowering the participants. Many of them reported that the program helped them changing their way of thinking by recognizing and modifying unhelpful thoughts.

*«The program gave me a good structure to think about how to approach difficult situations at home and also other things. It gave me a good structure to guide my thoughts, just the basic structure for difficult situations, emotions (…), and me, I had enough structure to hook onto and that immediately meant something to me.»*

Working on unhelpful thoughts also enabled them to see their situation in a less dramatic light and to manage negative emotions.

*« I learned to manage my behavior. I was, before I came, more impatient, I was more aggressive. I am less [aggressive], a lot less. Well, it can still happen at times when I am very tired […], but I am a lot less aggressive with him now, because before I blew up at every moment. Well anyway, now I have learned [to manage my behavior], this is positive. »*

The importance of taking some distance and taking better care of themselves were mentioned as helpful insights gained during the program.

*«[…] and also to be able to change the things a bit for me. To think a bit more about myself. Doing activities, going to the cinema with my friends a bit more often, to the theatre, and taking some time for myself, that is very important.»*

A deeper understanding of the disease and its symptoms empowered the participants to have more patience, to adapt their communication and to cope better with difficult behaviors of the person with dementia.

*«To say, ok: what do I do? I know he is ill, I know that he doesn’t have the capacity anymore, so it is not about me. I have to understand that. It [the program] brought greater understanding about the attitude of my husband [and] his behavior.»*

Information about, and contact details for diverse support organizations were experienced as helpful and facilitating access to support.

*«Then you recognize some openings for getting help. We know where to call, and who to turn to when something happens. This has been very positive.»*

## Discussion

This study aimed at 1) assessing whether the program “Learning to feel better… and help better” could be implemented in French-speaking Switzerland, 2) testing if the effects documented by the developers could be replicated in this new context, and 3) exploring the participants’ use of the trained strategies in daily life. Globally, feasibility was very good except for recruitment, and effects were positive and substantial, thus encouraging further dissemination of the program. The group format was a strong asset according to participants, and the use of reframing progressed the most over the course of the intervention.

Regarding feasibility, our qualitative and quantitative results converged towards a very good acceptance of the program and a strong implication of the participants. Recruitment was however challenging. The main barrier was the duration of the program, which discouraged many ICD from participating, as it was too demanding for them, particularly for older persons and those working full-time. Although underuse of diverse support options is very common among ICD and not specific to this program [27], we plan to further explore our recruitment challenges using interviews of the health care professionals and volunteers involved in this process. This information will help use revise the program and/or the recruitment strategy accordingly. Although the dropout rate was moderate, it also raises some issues. Dropouts notably occurred throughout the study and were in nearly all cases related to critical events affecting the ICD or the person with dementia. They therefore seem to be related to the vulnerability of older persons with dementia combined to the heightened risk of health challenges among ICD e.g. [5, 10], rather than to a lack of satisfaction with the program. In this line, reducing the duration of the program could decrease the dropout rate. However, compared to completers, the ICD who dropped out scored more positive at baseline on average (e.g. lower burden, less distress, more self-efficacy). Although these differences were not significant – which is not surprising given our small sample size – they could point to a selection bias towards an under-representation of less burdened individuals in our results. Replication in a larger sample should tell whether less burdened ICD are more likely to drop out from this program or whether this observation was a mere artifact, as is common in small samples.

Regarding effects, the program resulted in substantial improvements in burden, psychological distress and self-efficacy. As in the developers’ study [16] conducted in a highly similar sample, the intervention increased ICD quality of life. However the original randomized control trial had smaller effect sizes and found significant effects on different outcomes, namely the frequency of problem behavior of the person with dementia and the associated distress in the ICD. This discrepancy may be related to differences in study design, as a control group takes into account the effects of disease progression, which was not possible in our case. The high self-efficacy observed at the beginning of the program was very similar to the scores in the original validation study of the program (on a 0 to 100 scale, for the control and intervention group respectively, mean = 69.8 and 77.7, SD = 19.4 and 16.7; [16]). These observations indicate that participants in both studies had substantial resources, which is in line with feedbacks from the field professionals supporting us in the recruitment process, who noted that this program requires a significant involvement. Such a program may therefore not cater the primary needs of the most vulnerable ICD, such as those who are exhausted and may benefit in the first line from respite. Additional research is required to clarify the best timing for this intervention and its efficacy according to ICD characteristics. Our modest sample size and the diagnosis of dementia based on ICD report further limit the reliability of our results, which need confirmation within a large-scale randomized control trial including an external assessment of the diagnosis.

The qualitative results emphasized the benefits of a program offered in a group format, in line with results of a meta-analysis showing that psycho-educative interventions in a group format significantly reduced depression in ICD while individual ones did not [28]. Our participants reported reductions in loneliness and social isolation as they felt understood by their peers and could build new strong social bonds, in line with typical positive effects of support groups [15]. Yet, informal contacts with some participants after the end of the program suggested that embedding such a support-group component within a psycho-educational approach made a qualitative difference: Among those who joined the typical local support groups afterwards, many were disappointed about the stronger focus on expressing (negative) emotions than on solving problems, which they experienced as depressing. Further research assessing interventions’ efficacy in the area of ICD support should provide more information on the characteristics of the intervention to help disentangle the impact of diverse components.

Openly sharing experiences also facilitated social comparison and anticipation, having both negative and positive aspects. On the positive side, social comparison encouraged participants in their way of handling some situations and invited them to try new options by providing positive examples of management. Hearing others’ experiences also helped them get prepared for new challenges in their caregiving. On the negative side, participants were confronted with the painful experiences of others and detailed descriptions of the disease-related decline, which generated strong negative feelings in some of them, for a limited time. These feedbacks highlight some other processes at play beyond the training of coping strategies, many of them related to the complex mourning associated with dementia caregiving [29].

Moreover, the present research used an innovative data collection method to document strategy use. Collecting such information in daily life regularly throughout the intervention proved feasible despite the chronic stress experienced by ICD. These data confirmed that participants were using more and more over time the strategies they learned, and showed that reframing progressed the most. This last finding is in line with the results of a meta-analysis of 11 interventions for ICD comprising this strategy, which documented improvements in anxiety, depression and subjective stress - although no systematic effect was found for coping, subjective burden, reactions to the behaviors of the person with dementia, or institutionalization [30]. Notably, our participants rated reframing as the most challenging strategy to apply, but their qualitative feedbacks underlined how positively this skill affected their caregiving experience by restoring some control over their negative emotions, particularly when facing challenging behaviors of their loved one with dementia. Taken together, these results suggests that learning to reframe one’s thoughts is both important and difficult to achieve, and therefore requires specific attention from intervention developers and implementers.

Our less clear results regarding the use of problem solving and support seeking could be due to our questions being too general, as suggested by the high rates of mastery observed already at intervention start. These questions may have failed to capture what our participants did express in their qualitative feedbacks, about having found innovative solutions to their problems, and knowing better where and how to obtain the support they need. Our findings may also be limited by the lack of follow-up data, as some strategies take time to bear fruits, particularly in the area of support seeking. For example, organizing a day-care center for the person with dementia often spans over weeks between the first contact with the institution and the establishment of an adequate routine for all the persons involved. In the same line, managing the difficult behaviors of persons with dementia remains one of the most challenging caregiving task [31]. Therefore, ICDs may require more time to identify the triggers, as well as to develop and test new strategies, so that effects in this area may be better observed at follow-up.

## Conclusion

This study expands on the original one conducted by the developers of the program “Learning to feel better… and help better” in French-speaking Canada, by showing the feasibility and the very promising effects of this program in two regions of French-speaking Switzerland. The original efforts of our study to document the process of the intervention by measuring strategy use in daily life also pave the way for further research aiming to clarify the (most) active components in supporting ICDs. Our next challenge will be to shorten this program and to improve our recruitment procedure in order to make this helpful program accessible to more informal caregivers of persons with dementia.

## Abbreviations

IC: Informal caregivers
ICD: Informal caregivers of the person with dementia
MBP: Memory and behavioral problems
SD: Standard deviation
TIDieR: Template for Intervention Description and Replication
